# Type 2 diabetes in children, adolescents and young adults: association of birth weight, gestational age and metabolic syndrome

**DOI:** 10.1186/1758-5996-7-S1-A152

**Published:** 2015-11-11

**Authors:** Adriana Fornari, Suzana Coelho de Lavigne, Gislaine Vissoky Cé, Cesar Geremia, Siciane Grassiolli, Winston Boff, Mariana Gassen Santos, Balduino Tschiedel, Marcia Khaled Puñales Coutinho, Márjori da Silva Marroni

**Affiliations:** 1 Instituto da Criança com Diabetes, Porto Alegre, Brazil

## Background

The frequency of type 2 diabetes mellitus (T2D) in children and adolescents is increasing worldwide mainly due to the global epidemic of obesity in childhood. Environmental and genetic factors are being implicated in the pathogenesis of obesity and those who were born large (LGA) or small (SGA) for gestational age are at increased risk of developing obesity and metabolic syndrome (MS).

## Objective

The aim of the study was to evaluate the birth weight, gestational age and the prevalence of MS in T2D in youth.

## Material and methods

The study enrolled 65 subjects diagnosed as T2D up to 21 yrs. of age from a cohort that began in 2004 in a reference center. A cross-sectional study was conducted to obtain clinical and laboratory data from chart reviews. Body mass index (BMI) was defined according to World Health Organization (WHO) or National Center for Health Statistics (NCHS), adjusted for age and gender, and MS, to International Diabetes Federation (IDF) criteria.

## Results

Sixty five patients were included, with mean age (yrs.) of 14.5±3.1 at T2D diagnosis, diabetes duration of 5.7±4.1 (median 4.8) and 17.6±4.5 at baseline analysis. Among subjects, 65.2% were female, 59.1% white, 48.3% were born appropriate for gestational age (AGA), 34.5% SGA and 17.2% LGA. At diagnosis, 71.2% had acanthosis nigricans and 4.5% developed ketoacidosis (negative autoantibodies). Familial history of T2D was present in 88.7% (27.4% had familial coronary artery disease). Severe degrees of obesity (class II and III) were found in 58.6% of all sample and MS in 76,6%. Both were more frequent in those who were born LGA as compared to being born AGA (obesity: 80.0 vs 50.0%, p=0.099 and MS: 100.0 vs 66.7%, p=0.036) and no difference was found between SGA and AGA (obesity: 60.0 vs 50.0%, p=0.493 and MS: 80.0 vs 66.7%, p=0,312). The prevalence of the MS components was: 89.1% obesity (waist circumference), 80.6% abnormalities in lipid profile (high triglycerides or low high-density lipoprotein) and 46.1% hypertension. The BMI classification was: 1.5% normal, 26.2% overweight and 72.3% obesity (IMC: 31.9±6.8kg/m2, 24.6% with severe obesity).

## Conclusion

Our findings showed a high frequency of familial history of T2D, reflecting a genetic and environmental basis and also an increased prevalence in females, mostly adolescents. In addition, being born LGA predisposed to MS and severe obesity, emphasizing the importance of monitoring risk factors during gestation.

